# Outcomes of clinical decision support systems in real-world perioperative care: a systematic review and meta-analysis

**DOI:** 10.1097/JS9.0000000000001821

**Published:** 2024-07-22

**Authors:** Jianwen Cai, Peiyi Li, Weimin Li, Tao Zhu

**Affiliations:** a Department of Anesthesiology, West China Hospital, Sichuan University; b Laboratory of Anesthesia and Critical Care Medicine, National–Local Joint Engineering Research Centre of Translational Medicine of Anesthesiology, West China Hospital, Sichuan University; c The Research Units of West China (2018RU012) – Chinese Academy of Medical Sciences, West China Hospital, Sichuan University; d Department of Respiratory and Critical Care Medicine, West China Hospital, Sichuan University; e Institute of Respiratory Health, Frontiers Science Center for Disease-related Molecular Network, West China Hospital, Sichuan University; f State Key Laboratory of Respiratory Health and Multimorbidity, West China Hospital, Sichuan University, Chengdu, Sichuan, People’s Republic of China

**Keywords:** clinical decision support systems, decision-making efficiency, healthcare quality, meta-analysis, perioperative care, systematic review

## Abstract

**Background:**

Although clinical decision support systems (CDSS) have been developed to enhance the quality and efficiency of surgeries, little is known regarding the practical effects in real-world perioperative care.

**Objective:**

To systematically review and meta-analyze the current impact of CDSS on various aspects of perioperative care, providing evidence support for future research on CDSS development and clinical implementation.

**Methods:**

This systematic review and meta-analysis followed the Cochrane Handbook and PRISMA statement guidelines, searching databases up to 2 February 2024, including MEDLINE, PubMed, Embase, Cochrane, and Web of Science. It included studies on the effectiveness of CDSS in assisting perioperative decision-making, involving anesthesiologists, doctors, or surgical patients, and reporting at least one outcome such as complications, mortality, length of stay, compliance, or cost.

**Results:**

Forty studies met inclusion criteria, analyzing outcomes from 408 357 participants, predominantly in developed countries. Most perioperative CDSS use was associated with improved guideline adherence, decreased medication errors, and some improvements in patient safety measures such as reduced postoperative nausea and vomiting and myocardial injury. However, reported results varied widely, and no significant improvement in postoperative mortality was observed.

**Conclusion:**

The preliminary findings of this review offer an overview of the potential use of CDSS in real-world perioperative situations to enhance patient and anesthesiologist outcomes, but further researches with broader outcome dimensions, involving more stakeholders, and with longer follow-up periods are warranted for the critical evaluation of CDSS and then in better facilitate clinical adoption.

## Introduction

Highlights

- CDSS improves guideline adherence and reduces medication errors.
- Mixed results on patient safety improvements; no mortality reduction.
- Varied impacts on postoperative outcomes like nausea and myocardial injury.
- Studies predominantly from developed countries suggest limited global data.
- Further research is needed for broader outcome assessments and implementations.

The perioperative period, comprising the days and weeks immediately preceding and following surgical intervention, is a critical phase during surgical patient care^1^. Postoperative pulmonary complications, acute kidney injury, and major adverse cardiovascular and cerebrovascular events are frequent and serious issues that occur around the time of surgery. They lead to higher morbidity and mortality, longer hospital stays, and unfavorable long-term results, posing significant challenges for surgeons and anesthesiologists in providing perioperative care^2–4^. Estimated based on data from The Lancet Commission on Global Surgery, around 313 million surgical procedures are conducted globally a year, resulting in a minimum of 4.2 million deaths within 30 days post-surgery^5^. While actually, these deaths could have been prevented if perioperative adverse events and complications had been detected and handled in a timely and effective manner^1,6^.

In recent years, the increasing use of electronic health record systems and networked devices has sparked a growing interest in creating clinical decision support systems for medical environments^7^. Clinical decision support systems (CDSS) are computerized systems with relevant clinical knowledge and information that aim to enhance decision-making efficiency through human–computer interaction. CDSS are designed to offer many tasks like diagnosis, alert systems, prescription assistance, drug monitoring, and illness management. These features have the potential to enhance medical quality and patient outcomes^8,9^. However, the previously notable setbacks have also shown us that CDSS are not without risks^10^. Models derived from extensive datasets with high accuracy, often with an AUROC >0.8, may exhibit poor performance during external validation and in aiding clinical decisions, thereby jeopardizing patient safety^11^.

CDSS also hold great promise for use in the perioperative field. Research on advanced CDSS in the perioperative phase has lately expanded to cover preoperative risk assessment, intraoperative vital signs monitoring, postoperative problems prediction, and other related areas^12^. However, the perioperative procedure is dynamically changing, and the professionals involved are also complicated, while decision-making at this time can be life-threatening, which poses multiple obstacles to the application of CDSS in real clinical scenarios^13^. Research has shown that utilizing a prejudiced CDSS led to a 9.1 percentage point reduction in the accuracy of decision-making, potentially resulting in erroneous treatment^14^. On the other hand, the upfront expenses for implementing a new perioperative CDSS system can be substantial, and the ongoing maintenance costs also have to be taken into account. Therefore, it is important to evaluate the cost-effectiveness of utilizing the perioperative CDSS program^15^. Although a mass of perioperative CDSS have been developed and many reviews have outlined the benefits of CDSS, it is still uncertain whether and to what degree CDSS could truly have an expected impact and warrant implementation during the perioperative period^16–18^.

CDSS have been shown to be capable in the development stage of a positive impact on quality and efficiency of decision-making; however, experience shows that these benefits are not self-evident; the real effect of CDSS will definitely be influenced by various factors in real-world perioperative care rather be AUROC itself. Understanding the actual clinical application effects of existing CDSS plays a decisive role in the subsequent application strategies and improvement of CDSS. Thus, we conducted a systematic review and meta-analysis, aiming to comprehensively assess the current impact of CDSS in all aspects of the perioperative period so as to provide evidence support for future research and clinical practice.

## Methods

This systematic review and meta-analysis were reported in accordance with PRISMA (Preferred Reporting Items for Systematic Reviews and Meta-Analyses) and AMSTAR (Assessing the methodological quality of systematic reviews) Guidelines (High quality)^19,20^, as detailed in Supplemental Table 1 (Supplemental Digital Content 1, http://links.lww.com/JS9/D163, Supplemental Digital Content 2, http://links.lww.com/JS9/D164) and Supplemental Table 2 (Supplemental Digital Content 1, http://links.lww.com/JS9/D163). The study has been registered with the International Prospective Register of Systematic Reviews (PROSPERO).

### Search strategy

The literature search was conducted from database inception to 2 February 2024 on five electronic databases: MEDLINE, PubMed, Embase, Cochrane, and Web of Science. For identifying medical literature related to intervention (CDSS) in clinical settings (perioperative), medical subject headings (MeSH) terms (Decision Support Systems, Management [Mesh], Decision Support Systems, Clinical [MeSH Terms], anesthesia [MeSH Terms] and perioperative [MeSH Terms]) and keywords were used. The details of the complete database search strategy are provided in Supplemental Table 3 (Supplemental Digital Content 1, http://links.lww.com/JS9/D163).

### Study selection

Two reviewers jointly developed inclusion and exclusion criteria. After removing duplicates, two reviewers independently selected articles based on their titles and abstracts and then reviewed the entire text to select articles that met the criteria. At any stage, discrepancies are resolved by consensus after discussion among all authors. Studies were included when they met the following criteria: studies on the effectiveness of CDSS in assisting perioperative decision-making; studies involved anesthetists; certified registered nurse anesthetists or surgical patients; studies had to report at least one outcome (complications, mortality, length of stay, compliance or cost expenses, etc.). Studies were excluded when they met the following criteria: studies that did not use perioperative CDSS or CDSS does not have decision-making ability; studies focused on development for CDSS models/systems; reviews; abstracts; correspondence; case reports; non-English studies; studies not available in full text.

### Data extraction and quality assessment

All study data were independently extracted by two reviewers, and any differences were resolved through discussion among all authors. For each included study, we extracted the following information: author, year of publication, research design, study subjects and cohort size, intervention (CDSS type), outcome, etc.

All studies included in this review have assessed their methodological quality. For randomized controlled trial (RCT), we used the Risk of Bias (RoB) tool 2 to assess the risk of bias, in which bias is assessed as a judgment (high, low, or unclear) for individual elements from five domains (selection, performance, attrition, reporting, and other)^21^. For non-randomized studies, we used the Newcastle–Ottawa Scale (NOS) to assess the quality, in which a study is judged on three broad perspectives: the selection of the study groups; the comparability of the groups; and the ascertainment of either the exposure or outcome of interest for case–control or cohort studies, respectively^22^.

### Data synthesis and analysis

We classify all studies according to CDSS functions and benefits (patient safety, clinical management, drug consumption, user acceptance, and cost containment). When three or more studies report the same outcome, we conduct quantitative analysis, use forest charts to illustrate the results, and select the odds ratio (OR) or mean deviation (MD) and its 95% CI as the effect indicators. After extracting effect indicators, we use the chi-square test (*χ* ^2^ test) to perform heterogeneity analysis, while quantitatively assessing the magnitude of heterogeneity in conjunction with *I* ^2^. If *P*>0.1 and *I* ^2^<50%, it can be considered that there is homogeneity among different studies, and a fixed effects model mode is adopted. If *P*≤0.1 and *I* ^2^≥50%, it can be considered that there is heterogeneity among studies, and a random effects model is adopted. If the heterogeneity is significant, further sensitivity analysis will be performed. For other identified studies, due to differences in sample size, CDSS type, and outcome indicators, intervention measures and outcome measurements are highly heterogeneous, so we use qualitative analysis to provide a narrative summary. All statistical analyses are performed by using Revman 5.3.

## Results

### Study selection

**Figure 1:**
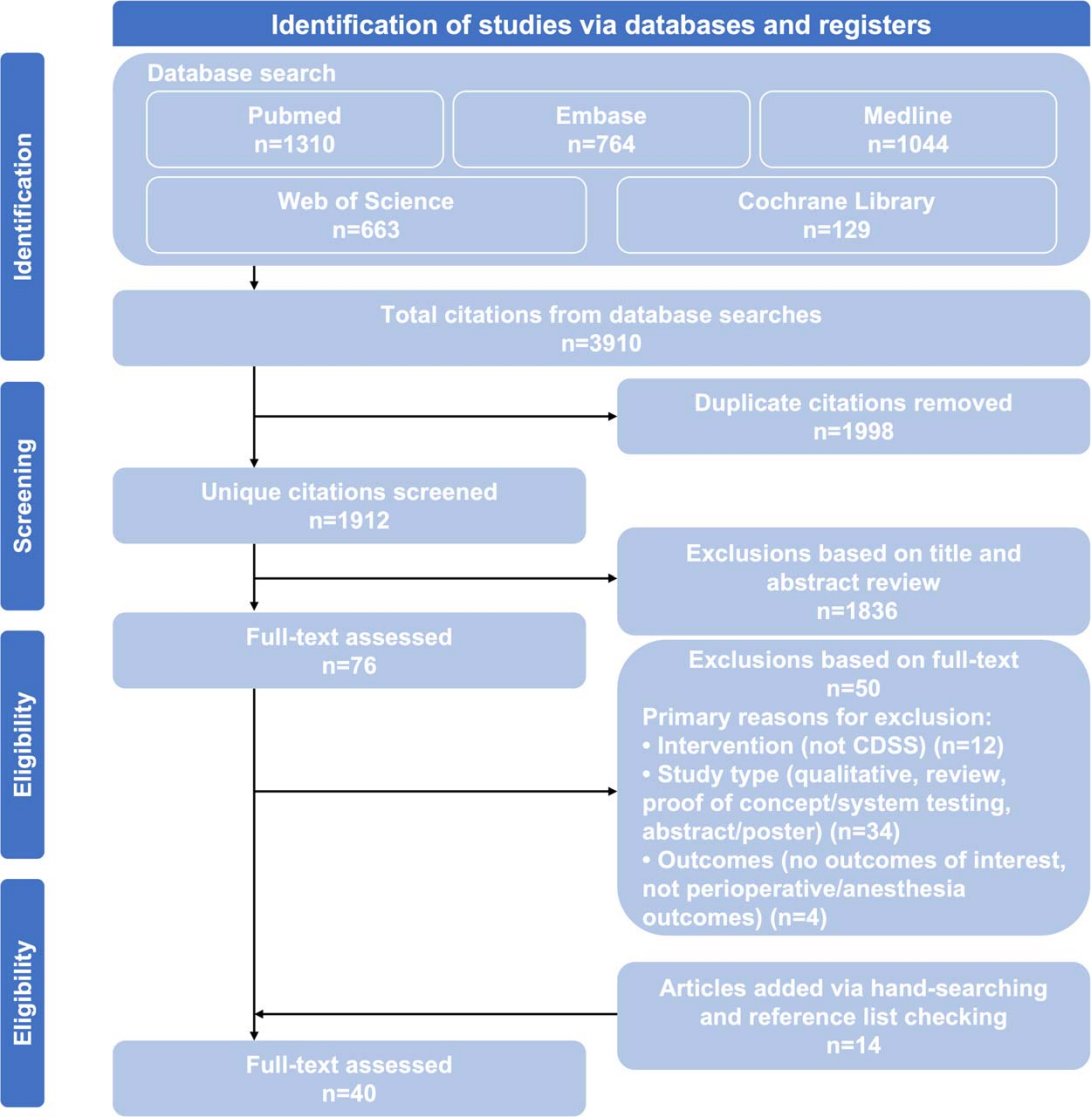
PRISMA flow diagram. PRISMA, Preferred Reporting Items for Systematic Reviews and Meta-Analysis.

Our search identified 3910 citations from five databases, of which 1998 were excluded due to duplication, 1836 were excluded after screening the title and abstract, and an additional 76 on full-text review, yielding a total of 40 studies that met all inclusion criteria (Fig. 1).

### Study characteristic

Characteristics of the included studies are presented in Table 1. All 40 included trials were published from 2010 to 2024. Thirty (75.0%) studies were conducted in the United States, three (7.5%) in France, two (5.0%) in the Netherlands, and one (2.5%) each in Germany, Brazil, Canada, Denmark, and Spain. For the study design, most of the included studies used cohort studies, including 17 (42.5%) prospective cohort studies, 10 (25.0%) retrospective cohort studies, and others were 12 (30.0%) randomized controlled trials and 1 (2.5%) case–control study. In total, 408 357 participants were analyzed, of which 407 692 (99.8%) participants were patients and 665 (0.2%) were anesthesiologists. Surgical services included general, ambulatory, urology, neurosurgery, orthopedic, laparoscopic, abdominal, trauma, cardiothoracic, ophthalmic, gynecology, plastics, breast, and vascular surgery.

**Table 1:** Study characteristics and results (*n*=40).

First author, year, country	Study design	Number of participants (patients or anesthesiologists)	Type of surgical procedure	Type of CDSS intervention	Outcome	Results
Ali, U, 2020, Canada^41^	Retrospective cohort	1127 patients (pediatric)	Ophthalmic surgery	A ‘strabismus macro’ automatically prepopulated the electronic anesthetic record with recommended bundle medications and delivered prompts and reminders for their use in all strabismus surgeries	(1) Guideline adherence (2) Moderate to severe postoperative pain (3) PACU stay	(1) Increased to 78.7% 2.21% in the CDSS group; 47.3% in the control group (3) No association: 95% CI, −3.86 to 8.86; *P*=0.439
Colletti, A.A., 2019, USA^42^	Retrospective cohort	39 patients (pediatric)	Neurosurgery	A novel, real-time algorithmic clinical decision support to guide pediatric TBI anesthesia care	(1) Hypocarbia (2) Hypotension (3) Hypothermia (4) Hyperthermia	(1) No association: 95% CI, −16.9 to 6.5; *P*=0.81 (2) No association: 95% CI, −2.4 to 3.6; *P*=0.74 (3) No association: 95% CI, −60.4 to 76.6; *P*=0.78 (4) No association: 95% CI, −6.0 to 3.8; *P*=0.09
Dherte, P.M., 2011, Brazil^43^	Case–control	64 patients (adult)	NR	A specific software integrating data monitored and indicating possible diagnosis according to specific parameters or association of parameters	Alarms	39 alarms in the smart alert group; 514 alarms in the control group
Gabel, E., 2019, USA^44^	Retrospective cohort	36 796 patients (pediatric and adult)	Multiple (Gynecology surgery, Neurosurgery, General surgery, Plastics surgery, Otolaryngology surgery, Laparoscopic surgery and others)	A real-time intraoperative checklist was reconfigured to give providers feedback as to whether the case complies with the PONV pathway	PONV	Positive association: 16.9% in the CDSS group, 95% CI,15.2–18.5%; *P*=0.007; 19.1% in the control group, 95% CI, 17.9–20.2%
Gopwani, S., 2023, USA^45^	Prospective cohort	176 patients (adult)	Major breast surgery	A CDSS includes data acquisition, data processing, and provider notification modules	(1) Guideline adherence (2) Administration of oral gabapentin (600 mg) (3) Administration of oral celebrex (400 mg) (4) Administration of scopolamine transdermal patch (5) Administration of ketamine (6) Administration of oral acetaminophen (7) PONV	1.44% in CDSS group; 16% in control group (*P*<0.001) 2.43% in CDSS group; 13% in control group (*P*<0.001) 3.35% in CDSS group; 16% in control group (*P*=0.006) 4.44% in CDSS group; 29% in control group (*P*=0.05) 5.7% in CDSS group; 4% in control group (*P*=0.35) 6.35% in CDSS group; 16% in control group (*P*=0.006) 7.14% in CDSS group; 8% in control group (*P*=0.31)
Gruss, C.L., 2023, USA^46^	Retrospective cohort	57 401 patients (adult)	NR	Feedback & CDS Recommendation, a daily case e-mail was sent to each provider preoperatively that recommended the number of PONV interventions based on a patient’s preoperative risk assessment	(1) Guideline adherence (2) PONV (3) PONV rescue medication administration	(1) Positive association: MD 5.5%, 95% CI, 4.2–6.4%; *P*<0.001 (2) No association: NR (3) Positive association: MD −8.7%; 95% CI, −10.2 to −7.1%; *P*<0.001
Gupta, R.K., 2014, USA^47^	Retrospective cohort	450 patients (adult)	Neurosurgery	An alert, in a computerized order entry system, when a practitioner attempts to order an anticoagulation medicine that matches the master list from the pharmacy on a patient with an existing epidural infusion order, that practitioner receives a warning alert before completing the order	Medication errors	11 events in the CDSS group; 26 events in the control group
Hand, W.R., 2014, USA^48^	Randomized controlled trial	111 anesthesiologists	N/A	The decision support tool (DST) containing the AHA/ACC evaluation and management guidelines was presented in electronic form on either an Apple iPad or iPhone	Guideline adherence	Positive association: MD 25%, *P*<0.0001
Joosten, A., 2021, France^49^	Randomized controlled trial	38 patients (adult)	Multiple (abdominal or orthopedic surgery)	A closed-loop vasopressor system was used to titrate vasopressor administration. A real-time clinical decision support system called ‘assisted fluid management’ was used to guide mini-fluid challenges	(1) Hypotension (2) Hypertension (3) Postoperative minor complications	(1) Positive association: 1.2% in CDSS group; 21.5% in control group; MD −21.1%; 95% CI −15.9 to −27.6; *P*<0.001 (2) Positive association: 2.5% in CDSS group; 12.9% in control group; *P*=0.001 (3) No association: 42% in CDSS group; 58% in control group; MD 16%; 95% CI −16 to 48; *P*=0.330
Kheterpal, S., 2018, USA^50^	Retrospective cohort	26 769 patients (adult)	Non-liver transplant surgery	AlertWatch OR, an FDA-cleared multifunction decision support system, is composed of real-time data extracted from physiologic monitors and data displayed in a readily identifiable schematic view of organ systems	(1) Hypotension (2) Myocardial injury (3) Stage 1 acute kidney injury (4) Stage 2 acute kidney injury (5) Mortality 30 days (6) Length of hospital stay	(1) Positive association: beta coefficient –0.29; 95% CI −0.30 to −0.27; *P*<0.001 (2) Positive association: AOR=0.68; 95% CI, 0.55–0.84; *P*<0.001 (3) No association: AOR=0.95; 95% CI, 0.88–1.03; *P*=0.24 (4) No association: AOR=0.91; 95% CI, 0.75–1.09; *P*=0.30 (5) No association: AOR=0.86; 95% CI, 0.69–1.06; *P*=0.16 (6) Positive association: beta coefficient −0.05; 95% CI −0.06 to −0.04; *P*<0.001
Kiatchai, T., 2017, USA^51^	Prospective cohort	22 patients (pediatric)	Neurosurgery	The anesthesia CDS, called Smart Anesthesia Manager (SAM), is a real-time CDS system developed by our institution. It works with an Anesthesia Information Management System (AIMS) to detect and alert ongoing clinical issues in real-time	User acceptance	88% of anesthesiologists reported that CDS helped with traumatic brain injury anesthesia care
Kooij, F.O., 2010, The Netherlands^52^	Prospective cohort	5652 patients (adult)	Non-cardiac surgery	A patient-specific decision support system (DSS), this system consisted of two different reminders that suggested administering PONV prophylaxis in case of impending nonadherence to the departmental guideline. The reminders were active both in the operating theater (operating theater reminder) and in the recovery room (recovery reminder)	Guideline adherence	Positive association: 79% in CDSS group; 39% in control group; *P*<0.001
Kooij, F.O., 2012, The Netherlands^53^	Prospective cohort	2662 patients (adult)	Non-cardiac surgery	A decision support system (DSS) using patient-specific automated reminders was implemented, supporting the physicians in their decision to prescribe PONV prophylaxis in the preoperative screening clinic and reminding the anesthesia team to administer PONV prophylaxis in the operating theater	(1) PONV (2) Overall use of dexamethasone, granisetron, and metoclopramide (3) Overall use of droperidol (4) PONV prophylaxis to high-risk patients (5) PONV prophylaxis to low-risk patients	(1) Positive association: 23% in the CDSS group; 27% in the control group; *P*=0.01 (2) No association: NR (3) Positive association: 1% in the CDSS group; 2% in the control group; *P*<0.001 (4) 91% (dexamethasone) and 87% (granisetron) in CDSS group; 82% (dexamethasone) and 76% (granisetron) in the control group; both *P*<0.001 (5) 21% (dexamethasone) in CDSS group; 14% (dexamethasone) in control group; *P*<0.001; 21% (granisetron) in CDSS group; 15% (granisetron) in control group; *P*=0.002
Lakha, S., 2020, USA^54^	Retrospective cohort	50 029 patients (pediatric and adult)	General surgery	A decision-support alert system is used, and alerts are sent to operating room workstations using an in-house notification system developed internally and written in Python. A local application running on each workstation is used to display the alert	Guideline adherence	92.4% (95% CI, 91.4–93.4%) in the CDSS group; 84.4% (95% CI, 83.6–85.2%) in the control group
Li, G., 2020, USA^55^	Prospective cohort	3706 patients (pediatric and adult)	NR	Vanderbilt’s Perioperative Information Management System (VPIMS) CDS was designed to provide in-room pop-up prompts to the providers to measure the glucose for patients who had impaired glucose management Best Practice Advisory (BPA) CDS. Based on a similar principle, the new intraoperative glucose alert was designed to facilitate the maintenance of normoglycemia in at-risk patients using Epic’s integrated clinical decision support framework	(1) Hyperglycemia (2) Hypoglycemia	(1) Positive association: 5.2% in VPIMS CDS group; 10.4% in the control group; *P*<0.001 10.4% in the control group; 7.2% in BPA CDS group *P*=0.031 (2) No association: 1.2% in VPIMS CDS group; 1.1% in control group; *P*=0.754 1.1% in control group; 1.4% in BPA CDS group *P*=0.504
Lipps, J., 2017, USA^56^	Randomized controlled trial	30 anesthesiologists	Cardiac surgery	A digital cognitive aid (DcogA) that is automatically triggered by a set vital sign aberration	User acceptance	3.43 in CDSS group; 1.57 in control group; *P*<0.01
McCormick, P.J., 2016, USA^57^	Randomized controlled trial	19 092 patients (adult)	Non-cardiac surgery	An automated intraoperative decision support alert for double-low conditions, defined by MAP less than 75 mmHg and BIS less than 45, reduces 90-day all-cause mortality	(1) Mortality within 90 days (2) Mortality within 30 days (3) Excess length of stay	(1) No association: 1.4% in CDSS group; 1.3% in control group; *P*=0.301 (2) No association: 0.66% in CDSS group; 0.68% in control group; *P*=0.980 (3) No association: 24% in CDSS group; 24% in control group; *P*=0.788
McEvoy, M.D., 2016, USA^58^	Randomized controlled trial	259 anesthesiologists	Multiple (Colorectal surgery, General surgery, Gynecologic surgery, Neurosurgery, Orthopedics, Otolaryngology, Plastic surgery, Spinal surgery, Thoracic surgery, Transplant, Urology, Vascular surgery and other)	A smartphone-based electronic decision support tool (eDST), the eDST used in this study was programed on the iOS platform and presented on the Apple iPod Touch	Guideline adherence	Positive association: 92.4±6.6% in CDSS group; 68.0±15.8% in control group; *P*<0.001
Mendez, J.A., 2018, Spain^59^	Prospective cohort	81 patients (adult)	Ambulatory surgery	A fuzzy logic controller is a tool able to evaluate some input information in order to generate an appropriate output based on heuristic knowledge given by experts	Bispectral Index monitoring (target)	Positive association: 53.09% in CDSS group (SD, 12.02%; 95% CI, 49.34–56.83%); 37.62% in control group (SD, 15.94%; 95% CI, 32.44–42.78%); *P*<0.001
Nair, B.G., 2016, USA^60^	Prospective cohort	3189 patients (adult)	General surgery	A near real-time decision support software module that was developed with internal resources at the University of Washington. It works in conjunction with an Anesthesia Information Management System to detect ongoing issues related to quality of care, billing, and compliance	Guideline adherence	71.2% (hourly glucose measurements) in CDSS group; 52.6% (hourly glucose measurements) in control group; *P*<0.001; 24.4% (correct insulin doses) in CDSS group; 13.5% (correct insulin doses) in control group; *P*=0.002
Nair, B.G., 2014, USA^61^	Prospective cohort	34 045 patients (adult)	Non-cardiac surgery	A real-time decision support module was developed at the University of Washington to improve the quality of care, billing, and compliance. Clinical data from the AIMS database are extracted and analyzed by SAM to detect selected issues related to quality of care, billing, and compliance based on a set of decision rules	(1) Hypotension-High Minimum Alveolar Concentration (MAC) Episodes (2) Hypertension-Phenylephrine Episodes	(1) Positive association: 2.4% in CDSS group; 2.9% in control group; *P*=0.031 (2) No association: 6.3% in CDSS group; 7.6% in control group; *P*=0.47
Nair, B.G., 2013, USA^62^	Prospective cohort	12 000 patients (adult)	NR	A real-time decision support system that was developed using University of Washington resources. SAM extracts clinical data at frequent periods from the AIMS database to analyze and detect selected issues related to quality of care, billing, and compliance based on a set of decision rules	Non-Invasive Blood Pressure gap (incidences per 1000 cases)	Positive association: 6.7±2.0 in CDSS group; 15.7±4.5 in control group; *P*<0.001 (Non-Invasive Blood Pressure gap >15 min) No association: 209±27 in CDSS group; 223±16 in control group; *P*=0.14 (Non-Invasive Blood Pressure gap <15 min)
Nair, B.G., 2010, USA^63^	Prospective cohort	17 815 patients (adult)	NR	A real-time decision support system, Smart Anesthesia Messenger (SAM). SAM is a server-based application that analyzes AIMS data in near real time with a sampling period of 6 min	Guideline adherence	Positive association: 90.0%±2.9% in CDSS group; 99.3%±0.7 in control group; MD 9.3%; 95% CI, −10.8 to −7.8; *P*<0.001
Nair, B.G., 2013, USA^64^	Prospective cohort	7523 patients (adult)	NR	A real-time decision support tool that works alongside the AIMS. SAM was internally developed by the University of Washington to improve the quality of care, anesthesia professional services billing, and compliance	Drug consumption (sevoflurane, desflurane, isoflurane) (FGF, l/min)	Positive association: 2.10±1.12 in CDSS group; 1.60±1.01 in control group; *P*<0.001
Nellis, J.R., 2023, USA^65^	Retrospective cohort	3617 patients (adult)	Colorectal surgery	An integrated, algorithm-based clinical decision support tool autonomously identified patients at high risk for AKI and 30-day readmission	(1) Acute kidney injury (2) Readmission	(1) Positive association: 8.8% in CDSS group; 11.3% in control group; *P*=0.034 (2) Positive association: 8.9% in CDSS group; 12.5% in control group; *P*=0.022
Olmos, A.V., 2023, USA^66^	Prospective cohort	90 467 patients (pediatric and adult)	NR	A decision support tool was designed using the intraoperative best practice advisory (BPA) functionality, a feature of the Epic AIMS that uses rule-based processing of real-time data, including device values, to fire alerts with various, programmable behaviors	(1) Sevoflurane consumption (FGF, l/min) (2) Desflurane consumption (FGF, l/min)	(1) Positive association: −0.6; 95% CI, −0.6 to −0.6; *P*<0.0001 (2) Positive association: −0.2; 95% CI, −0.2 to −0.3; *P*<0.0001
Olsen, R.M., 2018, Denmark^67^	Prospective cohort	178 patients (adult)	NR	A random forest algorithm was chosen since it is an ensemble algorithm, which, in multiple applications, has been proven to have high performance. Moreover, it still performs well even in the case of missing data, which was the case for some modalities during shorter periods of time, as seen	(1) Early signs of deterioration missed (2) False alarm	(1) 11.7% in CDSS group; 43.9% in control group; 99% CI, 61–85% (2) 9.4% in CDSS group; 32.7% in control group; 99% CI, 77–93%
Parks, D.A., 2021, USA^68^	Prospective cohort	109 anesthesiologists	Non-cardiothoracic surgery and Non-neurosurgical surgery	A multidimensional DS system for intraoperative processes, which was designed to optimize attending workflow while surfacing clinical data required for patient care, regulatory requirements, and billing	Guideline adherence	(1) Positive association: 69%, OR = 1.69; 95% CI, 1.46–1.96; *P*<0.01
Pouliot, J.D., 2018, USA^69^	Retrospective cohort	376 patients (adult)	Multiple (Burn, Cardiovascular surgery, Gastrointestinal or genitourinary surgery, Oncology surgery, Orthopedic surgery, Thoracic surgery, Trauma and Other)	A computerized clinical decision support module was built within the computerized physician order entry (CPOE) system at the institution. The anesthesia epidural ordering module was initially implemented to standardize the ordering of epidural anesthesia and associated orders	(1) Improper medication administered (2) Respiratory depression (3) Hypotensive events	(1) No association: 6.3% in CDSS group; 13.3% in control group; *P*=0.054 (2) No association: 4.0% in CDSS group; 3.9% in control group; *P*=0.97 (3) No association: 20.7% in CDSS group; 23.6% in control group; *P*=0.49
Pregnall, A.M., 2022, USA^70^	Retrospective cohort	31 887 patients (adult)	NR	A dashboard for self-monitoring high-dose sugammadex administration using the anesthesia registry functionality of our electronic health record; A case-specific, e-mail-based feedback contains a reporting of the patient’s actual body weight and the computed adjusted body weight, with a reminder of the dosing recommendation	(1) High-dose sugammadex consumption (2) Cost saving	(1) 4.3% in dashboard CDSS group; 3.1% in e-mail-based feedback CDSS group; 12.3% in control group (2) $61 274.57 in CDSS group; $63 837.62 in control group
Rinehart, J., 2012, USA^71^	Prospective cohort	60 patients (adult)	Orthopedic surgery	An algorithm monitors the patient’s hemodynamic parameters [in this study, CO, heart rate (HR), and mean arterial pressure (MAP)] and uses this information to determine appropriate fluid administration	Fluid administration (Total fluid given, liter)	Positive association: 2.1 l in CDSS group; 1.9 l in control group; *P*<0.05
Shah, A.C., 2019, USA^72^	Prospective cohort	1847 patients (adult)	Nonobstetric surgery	A real-time decision support system, Smart Anesthesia Manager targets anesthesia care to improve the quality of care, billing, and compliance. Clinical data from the AIMS database are extracted and analyzed by SAM to detect selected issues related to quality of care, billing, and compliance based on a set of predefined decision rules. If issues are detected, the anesthesia provider is notified	(1) Guideline adherence (2) Cumulative postoperative opioid consumption	(1) Positive association: 85% in CDSS group; 59% in control group; OR=2.78; 95% CI, 1.73–4.49; *P*<0.001 (2) No association: 34 mg in CDSS group; 62 mg in control group; *P*=0.38
Shear, T.D., 2019, USA^73^	Randomized controlled trial	34 anesthesiologists	NR	A dynamic electronic cognitive aid with embedded clinical decision support (dCA) and a static cognitive aid (sCA) tool, Gaba defined cognitive aid as a term that encompasses a host of physical (or now virtual) items aimed to assist professionals in executing the complex decision making of diagnosis and therapy	Task performance	Positive association: 15.70±1.93 in CDSS group; 12.95±2.16 in control group; *P*<0.0001
St Pierre, M., 2017, Germany^74^	Randomized controlled trial	54 anesthesiologists	Obstetric surgery	Crisis-related cognitive aids (CA), commonly referred to as a ‘crisis checklist’, ‘emergency manual’, or ‘emergency quick reference guide’, provide prompts for and reviews of critical steps during time-sensitive high-stress situations. Their goal is to offset the large cognitive load involved in crisis management and to help translate best practices for patient care during acute events	(1) Guideline adherence (2) Task performance	(1) Positive association: 93% in CDSS group; 69% in control group; *P*<0.001 (2) Positive association: 87.5% in CDSS group; 59% in control group; *P*<0.001
Velagapudi, M., 2023, USA^75^	Prospective cohort	10 anesthesiologists	General surgery	The machine learning decision support model estimates peak intraoperative glucose levels based on preoperative information. The intent of this model is to help anesthesiologists predict which patients will develop hyperglycemia during surgery and thus proactively plan for treatment. Another model estimates postoperative opioid requirements using preoperative information to help anesthesiologists prepare for pain management after surgery	(1) Task performance (peak glucose levels, Anesthesiologists’ estimation accuracy %) (2) Task performance (postoperative opioid requirement, Anesthesiologists’ estimation accuracy %)	(1) Positive association: 84.7±11.5% in CDSS group; 79.0±13.7% in control group; *P*<0.001 (2) Positive association: 42% in CDSS group; 18% in control group; *P*<0.001
Wanderer, J.P., 2012, USA^76^	Randomized controlled trial	20 anesthesiologists	NR	Anesthesia information management systems (AIMS) facilitate support for real-time decisions, promote clinician communication, and increase revenue through timely billing	Documentation accuracy	(1) 92.4% in CDSS group; 85.1% in control group; MD, 7.3; 95% CI, 1.8–12.7
Wetmore, D., 2016, USA^77^	Randomized controlled trial	38 anesthesiologists	NR	Pre-Anesthetic Induction Patient Safety (PIPS) checklist, the checklist includes items such as verification of working suction, backup airway device availability, adequate pre-anesthetic fasting assessment and several other key components of pre-anesthetic preparation	Task performance (out of 22 points)	Positive association: 16.79 in CDSS group; 8.42 in control group; 95% CI, 5.85–9.85; *P*<0.01
Wissel, B.D., 2023, USA^78^	Randomized controlled trial	284 patients (pediatric)	Neurosurgery	A decision support system screened patients with epilepsy before they were scheduled to visit a neurologist and sent automated alerts to their providers if they were predicted to be a surgical candidate	Referral	Positive association: 9.8% in CDSS group; 3.1% in control group; AHR=3.21; 95% CI, 0.95–10.8; one-sided *P*=0.03
Zaouter, C., 2017, France^79^	Randomized controlled trial	150 patients (adult)	Orthopedic surgery	An automatic closed-loop delivery system for propofol sedation, the hybrid sedation system (HSS). The DSS incorporated into the HSS offers the possibility to detect respiratory and hemodynamic critical events via audio-visual alarms, giving simultaneously decisional aids	Bispectral Index monitoring (target)	Positive association: 49% in CDSS group; 26% in control group; *P*<0.0001
Zaouter, C., 2014, France^80^	Randomized controlled trial	150 patients (adult)	Orthopedic surgery	An automated, closed-loop drug delivery system for sedation used in conjunction with spinal analgesia. A closed-loop system is composed of a control variable, actuator, and controller	(1) Low SpO_2_ (alarms/h) (2) Low respiratory rate (alarms/h) (3) Low mean arterial pressure (alarms/h) (4) Low heart rate (alarms/h) (5) False alarms (alarms/h)	(1) Positive association:0.7±1.0 in CDSS group; 1.4±2.2 in control group; *P*=0.036 (2) No association: 3.0±3.0 in CDSS group; 3.0±3.6 in control group; NR (3) No association: 4.7±6.4 in CDSS group; 3.5±3.6 in control group; NR (4) No association: 0.3±0.7 in CDSS group; 0.4±0.4 in control group; NR (5) No association: 25 in CDSS group; 19 in control group; NR

AHR, adjusted hazard ratio; AOR, adjusted odds, ratio; FGF, fresh gas flow; MD, mean difference; N/A, not applicable; NR, not reported; OR, odds, ratio; PACU, Post-anesthesia Care Unit; PONV, postoperative nausea and vomiting.

### Risk of bias and quality of evidence

**Figure 2:**
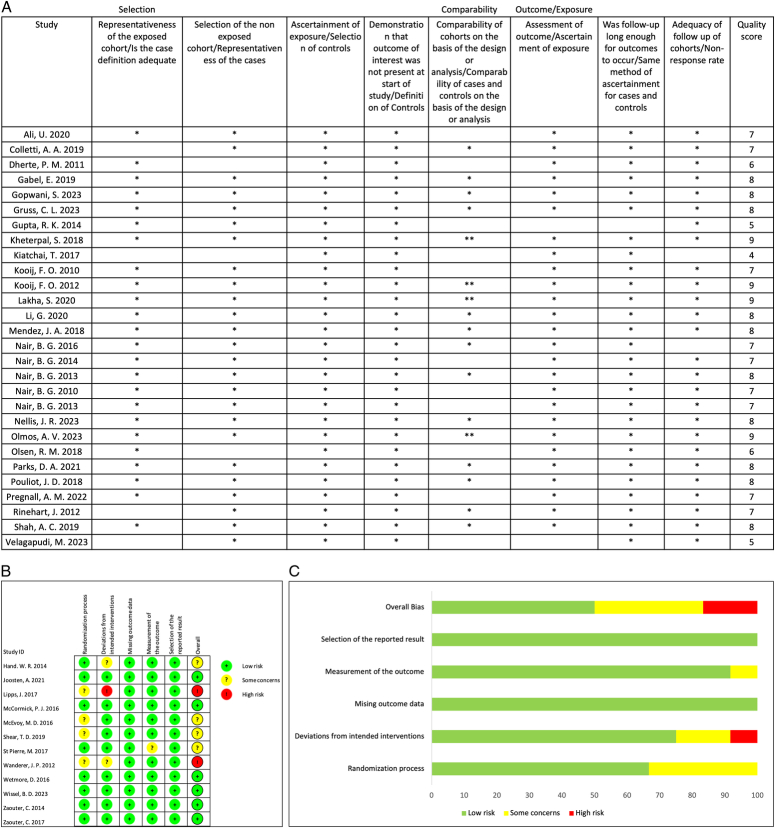
Risk of bias summary of included studies. (A) Risk of bias details of each study by Newcastle–Ottawa Scale (NOS) for non-randomized studies. (B) Risk of bias details of each study by Cochrane collaboration’s tool 2. (C) Risk of bias details by Cochrane collaboration’s tool 2.

For non-random controlled trial studies assessed by NOS, all studies had moderate to good quality reporting (Fig. 2A). For randomized controlled trials assessed by Rob 2, two studies were at a high risk of bias owing to deviations from the intended interventions and the randomization process, while four studies had moderate concerns, and six studies were at a low risk (Fig. 2B, C).

### Perioperative outcomes

#### Patient safety

**Figure 3:**
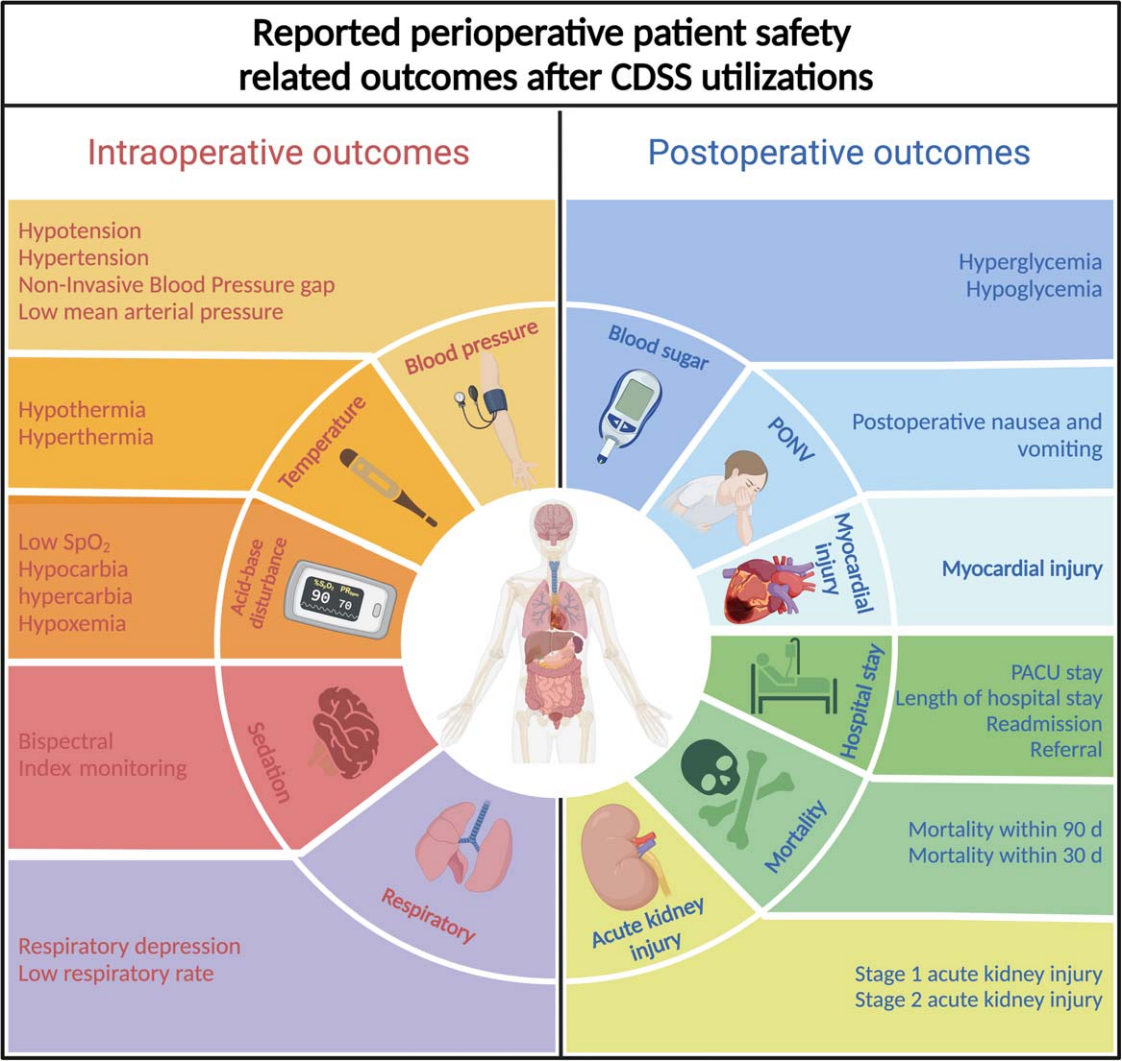
Reported perioperative patient safety-related outcomes after clinical decision support systems (CDSS) utilizations.

Nineteen studies reported (*n*_patients_=198 687) perioperative safety-related outcomes on intraoperative outcomes and postoperative outcomes, which are shown in Figure 3.

#### Intraoperative outcomes

Seven studies reported (*n*_patients_=73 417) blood pressure-related outcomes in multiple surgeries. Among seven, five studies (*n*_patients_=61 267) reported intraoperative hypotension events, three (*n*_patients_=60,852) of which found a significant association between the implementation of CDSS and the improvement in the reduction of different definitions of intraoperative hypotension events, another two studies (*n*_patients_=34 083) found the occurrence of intraoperative hypertension events decreased after the use of CDSS, but no statistical association between them. One study (*n*_patients_=12 000) found that CDSS could reduce the occurrence of extended non-invasive blood pressure gap exceeding 15 min (*P*<0.001), while another study (*n*_patients_=150) found that the number of low mean arterial pressure events/hour occurring was similar in both CDSS group and control group.

One study (*n*_patients_=39) examined the key performance indicators for traumatic brain injury anesthesia care using clinical decision support. They observed the reduction of median event duration of hypothermia (temperature <35.5°C) by 12% and hyperthermia (temperature >38.0°C) by 15% in the CDSS group, though none of these differences were statistically significant.

One study (*n*_patients_=64) investigated the role of CDSS in reducing false alarms in monitoring several events (hypoxemia, hypocapnia, hypercapnia, etc.) during general anesthesia, and a potential 92% reduction in invalid alarms was observed. Another study (*n*_patients_=150) found the incidence of oxygen desaturation (SpO_2_<92%) occurring per hour in orthopedic patients undergoing spinal analgesia with propofol sedation was significantly lower in the CDSS group (*P*=0.036).

Two studies (*n*_patients_=231) examined the performance of the automatic anesthesia sedation CDSS, which allowed for closed-loop delivery of propofol, in maintaining bispectral index (BIS): one study (*n*_patients_=150) reported clinical performance of sedation showing excellent control (BIS values fluctuation within 10%) in the CDSS group for a significantly longer period (49–26% in the control group, *P*<0.0001). Another study (*n*_patients_=81) found the percentage of time spent in the excellent bands (BIS values fluctuation within 10%) was significantly longer in the CDSS group (53.09%; SD, 12.02%; 95% CI, 49.34–56.83%) than in the control group (37.62%; SD, 15.94%; 95% CI, 32.44–42.78%) (*P*<0.001).

One study (*n*_patients_=376) found no statistically significant difference in intraoperative respiratory depression during epidural therapy (respiratory rate <8 breaths per minute) between the CDSS group and the control group (*P*=0.97). Similarly, another study (*n*_patients_=150) found the numbers of low respiratory rate events (respiratory rate <8 breaths per minute) occurring per hour in orthopedic patients under propofol sedation and spinal analgesia were similar for the CDSS group and the control group.

#### Postoperative outcomes

One study (*n*_patients_=3706) examined the incidence of hyperglycemia and hypoglycemia in a post-anesthesia nursing unit (PACU) during the replacement of VPIMS CDS with BPA CDS. Removing the CDSS temporarily led to a significant increase in hyperglycemia (from 5.2 to 10.4%, *P*<0.001) and a decrease from no CDS to BPA CDS (from 10.4 to 7.2%, *P*=0.031). No significant association was found in hypoglycemia (from 1.2 to 1.1%, *P*=0.754 and from 1.1 to 1.4%, *P*=0.504).

**Figure 4:**
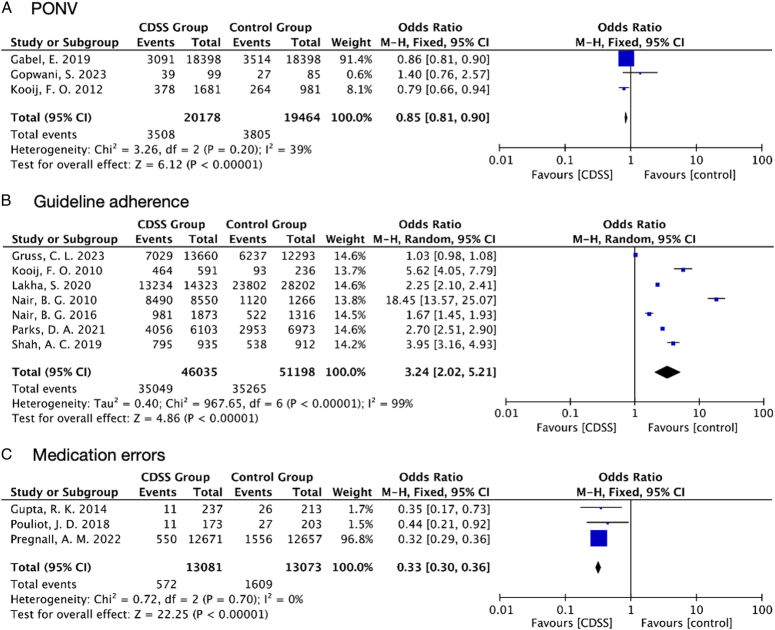
Forest plots from individual studies and meta-analysis for (A) postoperative nausea and vomiting (PONV), (B) Guideline adherence, and (C) Medication errors. M-H, Mantel–Haenszel.

Four studies (*n*_patients_=97 035) recorded the prevalence of postoperative nausea and vomiting (PONV) outcomes after the use of CDSS. Three of these studies (*n*_patients_=39 634) found CDSS performance was substantially linked to a decrease in the prevalence of PONV (OR 0.85; 95% CI, 0.81–0.90; *P*<0.00001; *I* ^2^=39%) (Fig. 4A). The one remaining study (*n*_patients_=57 401), which did not analyze the association of overall PONV rate change over the study periods, however, found there was no statistically or clinically significant reduction in the prevalence of PONV in the PACU with a combination of feedback and CDSS.

One study (*n*_patients_=26 769), which used combined multivariable analysis across three groups (CDSS group, historical control group, and parallel control group), found that CDSS use was an independent predictor and protective against postoperative myocardial injury (adjusted odds ratio 0.68; 95% CI, 0.55–0.84; *P*<0.001).

Three studies (*n*_patients_=46 988) examined the length of hospital stay and PACU stay; however, all investigations concluded that there was no significant correlation observed in either the CDSS group or the control group. One study (*n*_patients_=3617) discovered that using a risk-based management platform led to a 3.1% reduction in the rate of readmissions (from 12% to 8.9%, *P*=0.022). Another study (*n*_patients_=284) reported children who underwent epilepsy surgery whose perioperative provider received an alert from an automated decision support tool were more likely to be referred for a presurgical evaluation (3.1% vs. 9.8%; adjusted hazard ratio=3.21; 95% CI, 0.95–10.8; one-sided *P*=0.03).

Two studies (*n*_patients_=45 861) reported 30-day or 90-day all-cause in-hospital mortality but did not demonstrate statistically significant improvement in two types of mortality with CDSS application. Two studies (*n*_patients_=30 386) reported the incidence of postoperative AKI: one (*n*_patients_=3617) found applying a risk-based management platform was associated with a 2.5% decrease in the rate of AKI (11.3–8.8%, *P*=0.034), while another one (*n*_patients_=267 69) did not find any statistically significant improvement in stage 1 AKI (AOR=0.95; 95% CI, 0.88–1.03; *P*=0.24) or stage 2 AKI (AOR=0.91; 95% CI, 0.75–1.09; *P*=0.30).

### Clinical management

#### Guideline adherence

Data from seven studies (*n*_patients_=97 233) reporting the adherence to various perioperative guidelines on a composite outcome after utilization of CDSS, compliance rate, and percentage of compliance were evaluated. The results showed a considerably higher adherence to perioperative guidelines in the CDSS group [OR 3.24; 95% CI, 2.02–5.21; *P*<0.00001; *I* ^2^=99%] (Fig. 4B). A sensitivity analysis excluding each study individually revealed that no single study contributed to a substantial portion of the measured *I* ^2^ statistic. The single largest contribution was from the study by Gruss *et al*., which, when excluded, accounted for only 1% of heterogeneity. The remaining two studies included a similar topic of guideline adherence but did not have a comparable outcome or study design to include in the analysis. One study (*n*_anesthesiologists_=111) found using the CDSS resulted in a 25.0% improvement in adherence to guidelines (The 2007 American College of Cardiologists/American Heart Association Guidelines) compared to artificial memory. Another study (*n*_anesthesiologists_=18) identified nine evidence-based metrics of essential care from current guidelines (European Society of Cardiology Guidelines) and found the availability of the CDSS improved the overall task performance compared to memory alone (87.5% vs. 59.0%; *P*<0.001).

#### Task performance

Four studies (*n*_anesthesiologists_=341) evaluated the effectiveness of CDSS by comparing the scores or accuracy of anesthesiologists in solving and completing perioperative-related tasks with or without CDSS assistance. Two studies scored the task checklist performance of anesthesiologists separately by blinded raters; one study (*n*_anesthesiologists_=38) showed that there was a statistically significant difference in performance in pre-anesthetic induction evaluation (maximum score 22 points), and the CDSS group improved performance by 7.8 points (*P*<0.01), while another study (*n*_anesthesiologists_=34) found mean performance was statistically higher in the CDSS group versus the control group for total checklist (malignant hyperthermia, hyperkalemia, and ventricular fibrillation checklist) performance (15.70±1.93 vs. 12.95±2.16, *P*<0.0001, maximum score 22 points). One study (*n*_anesthesiologists_=259) scored the 20-question test performance of anesthesiology trainees and faculty in the evaluation and management of patients receiving antithrombotic or thrombolytic therapy and found the mean score was 92.4±6.6% in the CDSS group and 68.0±15.8% in the control group (*P*<0.001). Another study (*n*_anesthesiologists_=10) found the accuracy of peak glucose level estimates by the anesthesiologists increased from 79.0±13.7% without CDSS assistance to 84.7±11.5% (*P*<0.001) when CDSS were provided as reference.

### Medication administration

#### Medication errors

Three studies (*n*_patients_=26 154) reported on medication errors, the definition of which is similar, including preventable events that occur in any medication required for the perioperative care process, leading to improper medication or patient damage. The CDSS performance was significantly associated with reduced medication errors (OR 0.33; 95% CI, 0.30–0.36; *P*<0.00001; *I* ^2^=0%) (Fig. 4C).

#### Drug consumption

Six studies (*n*_patients_=102,508) reported drug consumption after using CDSS in the perioperative period. However, the type of drug included for the specific purpose varied among the studies, including analgesic, prophylactic, and inhalation anesthetics. Two studies (*n*_patients_=97 999) found 0.6 l/min reduced fresh gas flow of inhalation anesthetics after implementing CDSS. One study (*n*_patients_=2662), which patients were divided into a high-risk group and a low-risk group based on whether the risk factors related to PONV were greater than 3, reported on administration of prophylactic medication with significant increase achieved by CDSS employment in high-risk patients (82–91% for dexamethasone; 76–87% for granisetron) and decrease in low-risk patients (21–14% for dexamethasone; 21–15% for granisetron), the PONV risk was estimated by using Apfel’s simplified risk score. Another study (*n*_patients_=1847) reported on intraoperative and postoperative opioid use but found no statistically significant association between the CDSS intervention effect and the incidence or cumulative use of postoperative opioids after adjustment for preoperative time trends.

One study (*n*_patients_=60) compared the administration of fluid between anesthesiologists and a learning intravenous resuscitator (LIR) closed-loop system. The total amount of fluid administered was significantly higher in the LIR group than in the anesthesiologist-managed group (2172±323 ml vs. 1907±366 ml, *P*<0.05) and maintained more stable hemodynamics in patients.

### User interaction

Two studies (*n*_anesthesiologists_=52) used the Likert scale (1=strongly agree, 2=agree, 3=neutral, 4=disagree, and 5=strongly disagree) to evaluate anesthesiologists’ experience of CDSS use. Both studies showed that nearly all participants indicated CDSS had helped them to provide better perioperative care decision support to patients and increase their confidence in the management of intraoperative events.

One study (*n*_anesthesiologists_=20) examined the impact of a revised anesthesia information management system user interface, which provided continuous visual feedback by analyzing the anesthetic records for accuracy. The accuracy was determined by the percentage of correct data elements associated with each documentation. It reported that using the revised user interface could improve documentation accuracy from 85.1 to 92.4% (95% CI, 1.8–12.7), decrease the number of user interactions for intravenous (6.5 (95% CI, 2.9–10.1)) and airway documentation (16.1 (95% CI, 11.1–21.1)) and reduce airway documentation time by 30.5 s (95% CI, 8.5–52.4).

### Cost containment

Only one study (*n*_patients_=31 887) estimated potential weekly savings on the medical expenditure of sugammadex after implementing an automated system for displaying both a patient’s adjusted and actual body weight and an e-mail–feedback system to remind providers of guidelines using sugammadex. It reported that these associated initiatives lowered weekly expenditures on sugammadex to ~$61 274.57, assuming a cost of $119.69 per 200 mg vial of sugammadex, which meant an absolute weekly savings of $2563.05 and a relative reduction of 4% in the weekly expenditures on single-use sugammadex vials at the end of the study period.

## Discussion

This systematic review and meta-analysis summarized the current literature as of February 2024 on different types of CDSS implementation in varied surgeries and evaluated their effects on perioperative outcomes. The utilizing of CDSS in surgical patient care has been observed to be correlated with some improved prognoses, decreased occurrence of certain intraoperative adverse events and postoperative complications, as well as a reduction in some medication errors and timely administration of prophylactic medication. Anesthesiologists exhibited a preference for utilizing CDSS and showed enhanced performance in following guidelines and performing particular perioperative tasks when assisted by CDSS. Overall, despite certain negative results and omitted outcomes that were not evaluated, there were potential associations between the enhancement of reported perioperative outcomes and the implementation of CDSS in real-world settings.

Given how common CDSS are, it may be surprising that so little evidence has been evaluated to prove their impact on perioperative outcomes in real clinical scenarios until now^23^. Among the 3910 literature we have preliminarily searched, nearly 25% of them are related to the development of CDSS and internal validation in training datasets. When CDSS are applied into practice, it will face many obstacles. Without a systematic and scientific evaluation, the role of CDSS remains unknown to unsuspecting patients and professional users, which would lead to contrary effects as expected. Of the limited 40 studies we ultimately included, we found that 97.5% of the included studies came from developed countries, which meant they may have high-quality medical expertise and infrastructure in clinical settings. Accordingly, such systems remain under-explored and evaluated in low-resource settings, where they face a significant burden of a wide array of diseases, and the beneficial effects of CDSS implementation in these regions might be greater than in developed regions^24^. In addition, considering that interventions of CDSS are usually carried out in the entire population of medical institutions, it is difficult for most studies to seek suitable control groups to evaluate the effectiveness of CDSS, which means only including randomized trials might have excluded studies of innovations in CDSS when randomization was not acceptable or practicable to stakeholders^16^. Thus, we incorporated 28 rigorous non-randomized trials, including sequential trials, time-series studies, and so on, which would have provided a more thorough summary of current CDSS application research. Furthermore, the receiver of the CDSS project usually includes patients, so patient experience and feedback are also important in the implementation of CDSS. However, we were surprised to find that none of the studies included patient satisfaction as an evaluation indicator for CDSS application^25^. Although the application of CDSS may improve patient outcomes, this decision-making process is opaque. If anesthesiologists cannot explain the treatment plan or decision-making basis recommended by CDSS to patients, patients may have uncertainty about the decision-making process, which may lead to ethical hazards and unintended outcomes in practice.

Improving the outcomes of surgical patients is the primary intention of most development studies of perioperative CDSS. Although we found the use of CDSS was associated with an improvement in the decrease of some intraoperative adverse events and postoperative complications in the hospital, these positive results were not necessarily synonymous with an improvement in the patient’s prognosis. Mortality is the most concerning indicator for patients and a crucial endpoint for evaluating patient outcomes, but only a few studies have reported 30-day or 90-day mortality and found no statistically significant improvement, partly due to these outcomes being complex and associated with multiple etiologies such as the effect of the surgery quality, patient demographic, or comorbidities^26,27^. Additionally, the follow-up periods of included studies were insufficient to discover a significant number of positive outcomes to provide firm evidence; a longer time window (days out of the hospital at 1 year after surgery) may be required for long-term dynamic tracking of the true effectiveness of CDSS in a manner detectable at a population level^28^. Furthermore, standardized perioperative endpoint reporting is the cornerstone for outcomes assessment, but we found an inconsistency in the definitions of certain adverse events across various studies. The incidence of such incidents might decrease overall after adopting CDSS but would vary depending on different criteria, which complicates result interpretation due to various circumstances. These findings emphasized the importance of adopting a standardized set of criteria for defining and reporting intraoperative adverse events, as previously suggested^29,30^.

Guidelines adherence is a key factor in improving medical quality and patient care outcomes. However, compliance with guidelines in clinical practice is not ideal since the premise that professionals will actively read, assimilate, and execute novel guidelines has proven to be unrealistic^31^. In this review, we found that the application of CDSS can improve adherence to multiple guidelines and improve patient-related outcomes in the perioperative context. This is an inspiring result, but an important issue when trying to increase professionals’ adherence to guidelines is to understand the reasons why they have poor adherence to guidelines before using CDSS. Because, in fact, it cannot be ignored that CDSS are also a ‘black box,’ it is very dangerous for professionals to only follow CDSS’ instructions if they are unaware of the guidelines’ contexts^32^. CDSS may have a ‘carryover effect’ over time. Providers may become overly dependent or trusted on CDSS for a particular task, making them less capable of performing it in the event that they move to an environment without the CDSS^33^. Therefore, implementation of CDSS must occur alongside co-interventions such as education and training to synchronously influence providers’ behavior^10^.

CDSS have proven to be quite effective in lowering rates of medication errors by offering real-time alerts about drug interactions, incorrect dosage, and allergic responses. Additionally, an intelligent and automated trend in perioperative drug administration has emerged as a result of the recent use of AI algorithms in CDSS, which can improve patient safety by averting possible adverse medication events^34^. Nonetheless, the early strategy for ensuring patient safety in anesthesia was based on the individual, with clinicians assuming exclusive responsibility for maintaining their patients’ safety^35^. This change indicates that independent CDSS is gaining the ability to make decisions regarding the administration of medication in place of anesthesiologists. Is the CDSS function, on the one hand, strong enough to persuade anesthesiologists to accept the risk of treating probabilities? On the other hand, who bears the blame for unfavorable results – CDSS or anesthesiologists? All of these are unknowns that need to be explained by additional comparative research and ethical guidelines.

Alert fatigue is a common and inevitable phenomenon in the practical application of CDSS. The principle of CDSS generating alerts in decision support is related to its threshold setting. These alerts are intended to notify perioperative professionals of changes in patients’ physiological parameters or even potentially life-threatening conditions, but this does not mean that the threshold for most alerts is set to be urgent or harmful. As previous studies demonstrated, 95% of CDSS alarms may be inconsequential, and oftentimes, professionals disagree with or distrust alarms^36^. If too many insignificant alarms or CDSS recommendations are imposed on professionals, it may contribute to concealing the real effect of CDSS by human factors and then influence patient prognosis^37^. However, the included studies did not find evidence of alarm fatigue among professionals. Conversely, we found anesthesiologists have a high acceptance of CDSS, and CDSS could increase their confidence in the management of intraoperative events. This positive attitude toward CDSS intervention needs to be noted because included studies only evaluate the acceptance in the short-term application, or even the performance after one single attempt, without considering the potential alert fatigue that may gradually happen after long-term practical use. Meanwhile, this long-term alert fatigue may have a cumulative and deep-rooted effect; perioperative professionals may become desensitized from repeated exposure to a similar alert over time, and they would perform worse compared to the initial application and adopt a rejection mentality toward the application of new alerts^37^. Thus, an ongoing evaluation is required in the process of CDSS practice as well as adjustment of the functions, such as giving priority to alerts that are extremely significant or customizing alerts to particular specializations and severities^38^.

CDSS can be cost-effective for health systems by reducing medical expenditures directly related to patients, doctors, and medical institutions. On the one hand, for patients, the role of CDSS in their medical expenses is reflected in reducing the incidence of intraoperative adverse events and postoperative complications, thereby avoiding additional expenses caused by prolonged hospital stays. On the other hand, for physicians, the addition of CDSS in perioperative tasks may reduce their workload and improve their work efficiency, thereby saving them time costs and reducing the labor required for perioperative tasks. However, it is undeniable that putting CDSS intervention into practice needs a sizable initial investment as well as continuing maintenance expenditures^39,40^. As with previous reviews, we discovered that the poor quality of the reporting impeded the understanding of the economic findings^18^. Many research studies overlook economic issues in their design, and at the same time, the cost of starting CDSS and the expenses required for later maintenance were not reported, leading to a deficiency in cost-effectiveness outcomes. The cost of implementation is a key factor in clinical practice and may outweigh the benefits in patient care, and future trials should include more cost-effectiveness data to clearly express the value proposition of CDSS technologies.

Ultimately, we discovered during the evaluation process that in one instance, CDSS were utilized, but the patient-related outcomes worsened rather than improved when compared to normal treatment. Although the use of CDSS is expected to be viewed as an intervention that can enhance perioperative patient-related outcomes, it is crucial to consider whether the quality of CDSS are subpar and have negative or unexpected effects. This is due to the possibility that users may create workarounds in badly designed systems, such as submitting inaccurate or generic data, which compromises the knowledge base of CDSS^36^. On the other hand, the available data are also insufficient to say with certainty if the adoption of CDSS would have unfavorable impacts, which emphasizes the significance of carefully assessing these interventions in order to successfully handle implementation issues.

This study is the first systematic review and meta-analysis of the effect of CDSS on perioperative outcomes. The strengths of this review include a comprehensive literature search, which included an examination of major databases and an addition of hand-searching and reference list checking. Meanwhile, an assessment of risk of bias and quality was performed in each study, and it was found that all non-random controlled trials had moderate to good quality and that only two random controlled trials were at a high risk of bias. However, this review is not without limitations. Firstly, considering that these studies varied in design and involved diverse types of CDSS and surgical procedures, as well as different populations, our results should be interpreted with caution. Besides, due to the scarcity of data and different definitions and measures among similar outcomes, a meta-analysis could only be conducted on the incidence of PONV, guideline adherence, and medication errors. Furthermore, substantial heterogeneity was also observed in guideline adherence (*I* ^2^ was 99%), and even after sensitive analysis via repeated analyses with sequential exclusion of each study individually, the minimum *I* ^2^ value observed was 98%, suggesting that the studies may be heterogeneous at baseline. Additionally, this may be because of the different types of guidelines included in the studies and the varying degrees of difficulty in adhering to these guidelines.

## Conclusion

In conclusion, despite the existing scarcity of such studies, this systematic review and meta-analysis offers an overview of the potential use of CDSS in real-world perioperative situations to enhance patient and anesthesiologist outcomes. Although most of the results are positive, the interpretation and evaluation for such results still need to be given more attention in terms of outcome definitions, evaluation dimension, stakeholders, and follow-up period. It is imperative that more research be done to address critical questions: what support should perioperative CDSS provide for anesthesiologists and patients in decision-making, when and how it should be applied sustainably across surgical scenarios, and how to critically evaluate the implementation outcomes of perioperative CDSS in order to ensure the widespread clinical adoption of CDSS in the future.

## Ethical approval

This is a systematic review and meta-analysis of published data; thus, no ethical approval was required.

## Consent

This study is a systematic review and meta-analysis, not involving any patients or volunteers; thus, it does not require ethics committee approval and fully informed written consent.

## Source of funding

This study was funded by the National Natural Science Foundation of China (72204174 to PL, 91859203 to WL), the Science and Technology Project of Sichuan (2020YFG0473 to WL), the China Postdoctoral Science Foundation (2022M722262 to PL), the Postdoctoral Program of Sichuan University (2024SCU12026 to PL), the Postdoctoral Program of West China Hospital, Sichuan University (2023HXBH009 to PL), the 1·3·5 project for disciplines of excellence, West China Hospital, Sichuan University (ZYJC21008 to TZ), the Sichuan Province Natural Science Foundation of China (2023NSFSC0512 to TZ), and the CAMS Innovation Fund for Medical Sciences (2023-I2M-C&T-B-122 to TZ).

## Author contribution

J.C. and P.L.: study conception/design and data acquisition and analysis; J.C., P.L., W.L., and T.Z.: interpreting results; J.C.: initial drafting of the manuscript; P.L. and T.Z.: critical revision of the manuscript.

## Conflicts of interest disclosure

The authors declare that they have no conflicts of interest.

## Research registration unique identifying number (UIN)

1. Name of the registry: PROSPERO.
2. Unique identifying number or registration ID: CRD42023449940.
3. Hyperlink to your specific registration (must be publicly accessible and will be checked): https://www.crd.york.ac.uk/prospero/display_record.php?ID=CRD42023449940.

## Guarantor

Tao Zhu, MD, PhD, Department of Anesthesiology, West China Hospital, Sichuan University, Guo Xue Xiang 37, Chengdu 610041, Sichuan, People’s Republic of China; e-mail: 739501155@qq.com, Tel.: +86 028 85423593.

## Data availability statement

All data are available in the manuscript.

## Provenance and peer review

Not commissioned, externally peer-reviewed.

## Supplementary Material

SUPPLEMENTARY MATERIAL
