# Supplementary material for: Outcomes of clinical decision support systems in real-world perioperative care: a systematic review and meta-analysis
Source: Int J Surg. 2024 Jul 22;110(12):8057–72. doi: 10.1097/JS9.0000000000001821 (PMC11634111; doi:10.1097/JS9.0000000000001821)
Supplement: SUPPLEMENTARY MATERIAL [file js9-110-8057-s001.docx]

**ONLINE SUPPLEMENT with:**

**Outcomes of Clinical Decision Support Systems in Real-world Perioperative Care: A Systematic Review and Meta-analysis**

**Supplemental Table 1.** PRISMA Checklist

**Supplemental Table 2.** AMSTAR 2

**Supplemental Table 3.** Definite search

**Supplemental Text 1.** Reference List of Included Studies

**Supplemental Table 1.** PRISMA Checklist

| **Section and Topic** | **Item #** | **Checklist item** | **Location where item is reported** |
| --- | --- | --- | --- |
| **TITLE** | | |  |
| Title | 1 | Identify the report as a systematic review. | Title Page |
| **ABSTRACT** | | |  |
| Abstract | 2 | See the PRISMA 2020 for Abstracts checklist. | Page 1-2 |
| **INTRODUCTION** | | |  |
| Rationale | 3 | Describe the rationale for the review in the context of existing knowledge. | Page 3-4 |
| Objectives | 4 | Provide an explicit statement of the objective(s) or question(s) the review addresses. | Page 3-4 |
| **METHODS** | | |  |
| Eligibility criteria | 5 | Specify the inclusion and exclusion criteria for the review and how studies were grouped for the syntheses. | Page 5 |
| Information sources | 6 | Specify all databases, registers, websites, organisations, reference lists and other sources searched or consulted to identify studies. Specify the date when each source was last searched or consulted. | Page 4-5 |
| Search strategy | 7 | Present the full search strategies for all databases, registers and websites, including any filters and limits used. | Page 4-5, Supplement Table 3 |
| Selection process | 8 | Specify the methods used to decide whether a study met the inclusion criteria of the review, including how many reviewers screened each record and each report retrieved, whether they worked independently, and if applicable, details of automation tools used in the process. | Page 5 |
| Data collection process | 9 | Specify the methods used to collect data from reports, including how many reviewers collected data from each report, whether they worked independently, any processes for obtaining or confirming data from study investigators, and if applicable, details of automation tools used in the process. | Page 5-6 |
| Data items | 10a | List and define all outcomes for which data were sought. Specify whether all results that were compatible with each outcome domain in each study were sought (e.g. for all measures, time points, analyses), and if not, the methods used to decide which results to collect. | Page 5-6 |
|  | 10b | List and define all other variables for which data were sought (e.g. participant and intervention characteristics, funding sources). Describe any assumptions made about any missing or unclear information. | Page 5-6 |
| Study risk of bias assessment | 11 | Specify the methods used to assess risk of bias in the included studies, including details of the tool(s) used, how many reviewers assessed each study and whether they worked independently, and if applicable, details of automation tools used in the process. | Page 5-6 |
| Effect measures | 12 | Specify for each outcome the effect measure(s) (e.g. risk ratio, mean difference) used in the synthesis or presentation of results. | Page 6 |
| Synthesis methods | 13a | Describe the processes used to decide which studies were eligible for each synthesis (e.g. tabulating the study intervention characteristics and comparing against the planned groups for each synthesis (item #5)). | Page 6 |
|  | 13b | Describe any methods required to prepare the data for presentation or synthesis, such as handling of missing summary statistics, or data conversions. | Page 6 |
|  | 13c | Describe any methods used to tabulate or visually display results of individual studies and syntheses. | Page 6 |
|  | 13d | Describe any methods used to synthesize results and provide a rationale for the choice(s). If meta-analysis was performed, describe the model(s), method(s) to identify the presence and extent of statistical heterogeneity, and software package(s) used. | Page 6 |
|  | 13e | Describe any methods used to explore possible causes of heterogeneity among study results (e.g. subgroup analysis, meta-regression). | Page 6 |
|  | 13f | Describe any sensitivity analyses conducted to assess robustness of the synthesized results. | Page 6 |
| Reporting bias assessment | 14 | Describe any methods used to assess risk of bias due to missing results in a synthesis (arising from reporting biases). | Page 6 |
| Certainty assessment | 15 | Describe any methods used to assess certainty (or confidence) in the body of evidence for an outcome. | N/A |
| **RESULTS** | | |  |
| Study selection | 16a | Describe the results of the search and selection process, from the number of records identified in the search to the number of studies included in the review, ideally using a flow diagram. | Page 6, Figure 1 |
|  | 16b | Cite studies that might appear to meet the inclusion criteria, but which were excluded, and explain why they were excluded. | Page 6, Figure 1 |
| Study characteristics | 17 | Cite each included study and present its characteristics. | Table 1 |
| Risk of bias in studies | 18 | Present assessments of risk of bias for each included study. | Page 7, Figure 2 |
| Results of individual studies | 19 | For all outcomes, present, for each study: (a) summary statistics for each group (where appropriate) and (b) an effect estimate and its precision (e.g. confidence/credible interval), ideally using structured tables or plots. | Table 1,Figure 4 |
| Results of syntheses | 20a | For each synthesis, briefly summarise the characteristics and risk of bias among contributing studies. | Table 1, Figure 2 |
|  | 20b | Present results of all statistical syntheses conducted. If meta-analysis was done, present for each the summary estimate and its precision (e.g. confidence/credible interval) and measures of statistical heterogeneity. If comparing groups, describe the direction of the effect. | Page 9-11 |
|  | 20c | Present results of all investigations of possible causes of heterogeneity among study results. | Page 10 |
|  | 20d | Present results of all sensitivity analyses conducted to assess the robustness of the synthesized results. | Page 10 |
| Reporting biases | 21 | Present assessments of risk of bias due to missing results (arising from reporting biases) for each synthesis assessed. | N/A |
| Certainty of evidence | 22 | Present assessments of certainty (or confidence) in the body of evidence for each outcome assessed. | N/A |
| **DISCUSSION** | | |  |
| Discussion | 23a | Provide a general interpretation of the results in the context of other evidence. | Page 12-17 |
|  | 23b | Discuss any limitations of the evidence included in the review. | Page 12-17 |
|  | 23c | Discuss any limitations of the review processes used. | Page 17-18 |
|  | 23d | Discuss implications of the results for practice, policy, and future research. | Page 12-17 |
| **OTHER INFORMATION** | | |  |
| Registration and protocol | 24a | Provide registration information for the review, including register name and registration number, or state that the review was not registered. | Title Page |
|  | 24b | Indicate where the review protocol can be accessed, or state that a protocol was not prepared. | Title Page |
|  | 24c | Describe and explain any amendments to information provided at registration or in the protocol. | Title Page |
| Support | 25 | Describe sources of financial or non-financial support for the review, and the role of the funders or sponsors in the review. | Title Page |
| Competing interests | 26 | Declare any competing interests of review authors. | Title Page |
| Availability of data, code and other materials | 27 | Report which of the following are publicly available and where they can be found: template data collection forms; data extracted from included studies; data used for all analyses; analytic code; any other materials used in the review. | N/A |

**Supplemental Table 2.** AMSTAR 2

| **1. Did the research questions and inclusion criteria for the review include the components of PICO?** | | | | | | | | | |
| --- | --- | --- | --- | --- | --- | --- | --- | --- | --- |
| For Yes:  √ Population  √ Intervention  √ Comparator group  √ Outcome | | | Optional (recommended)   Timeframe for follow-up | | | √   | | Yes No |  |
| **2. Did the report of the review contain an explicit statement that the review methods were established prior to the conduct of the review and did the report justify any significant deviations from the protocol?** | | | | | | | | | |
|  | For Partial Yes:  The authors state that they had a written protocol or guide that included ALL the following:  √ review question(s)  √ a search strategy  √ inclusion/exclusion criteria  √ a risk of bias assessment | | For Yes:  As for partial yes, plus the protocol should be registered and should also have specified:   - a meta-analysis/synthesis plan, if appropriate, *and* - a plan for investigating causes of heterogeneity - justification for any deviations from the protocol | | | √     | | Yes Partial Yes No |  |
| **3. Did the review authors explain their selection of the study designs for inclusion in the review?** | | | | | | | | | |
|  | For Yes, the review should satisfy ONE of the following:   - *Explanation for* including only RCTs - OR *Explanation for* including only NRSI   √ OR *Explanation for* including both RCTs and NRSI | | | | | √   | | Yes No |  |
| **4. Did the review authors use a comprehensive literature search strategy?** | | | | | | | | | |
|  | For Partial Yes (all the following):  √ searched at least 2 databases (relevant to research question)  √ provided key word and/or search strategy  √ justified publication restrictions (e.g. language) | | For Yes, should also have (all the following):   - searched the reference lists / bibliographies of included studies - searched trial/study registries - included/consulted content experts in the field - where relevant, searched for grey literature - conducted search within 24 months of completion of the review | | |   √   | | Yes Partial Yes No |  |
|  | **5. Did the review authors perform study selection in duplicate?** | | | | |  | | |  |
|  | For Yes, either ONE of the following:  √ at least two reviewers independently agreed on selection of eligible studies and achieved consensus on which studies to include   - OR two reviewers selected a sample of eligible studies and achieved good agreement (at least 80 percent), with the remainder selected by one reviewer. | | | | | √   | | Yes No |  |
| **6. Did the review authors perform data extraction in duplicate?** | | | | | | | | | |
| For Yes, either ONE of the following:  √ at least two reviewers achieved consensus on which data to extract from included studies   - OR two reviewers extracted data from a sample of eligible studies and achieved good agreement (at least 80 percent), with the remainder extracted by one reviewer. | | | | | | | √ Yes   - No | | |
| **7. Did the review authors provide a list of excluded studies and justify the exclusions?** | | | | | | | | | |
|  | | For Partial Yes:  √ provided a list of all potentially relevant studies that were read  in full-text form but excluded from the review | For Yes, must also have:   Justified the exclusion from the review of each potentially relevant study | | | | - Yes   √ Partial Yes   - No | | |
| **8. Did the review authors describe the included studies in adequate detail?** | | | | | | | | | |
|  | | For Partial Yes (ALL the following):  √ described populations  √ described interventions  √ described comparators  √ described outcomes  √ described research designs | For Yes, should also have ALL the following:   - described population in detail - described intervention in detail (including doses where relevant) - described comparator in detail (including doses where relevant) - described study’s setting - timeframe for follow-up | | | | - Yes   √ Partial Yes   - No | | |
| **9. Did the review authors use a satisfactory technique for assessing the risk of bias (RoB) in individual studies that were included in the review?** | | | | | | | | | |
|  | | **RCTs**  For Partial Yes, must have assessed RoB from   - unconcealed allocation, *and* - lack of blinding of patients and assessors when assessing outcomes (unnecessary for objective outcomes such as all-   cause mortality) | For Yes, must also have assessed RoB from:  √ allocation sequence that was not truly random, *and*  √ selection of the reported result from among multiple measurements or analyses of a specified outcome | | | | √ Yes   - Partial Yes - No - Includes only NRSI | | |
|  | | **NRSI**  For Partial Yes, must have assessed RoB:   - from confounding, *and* - from selection bias   **10. Did the review authors report o** | For Yes, must also have assessed RoB:  √ methods used to ascertain exposures and outcomes, *and*  √ selection of the reported result from among multiple measurements or analyses of a specified outcome  **n the sources of funding for the studies inc** | | | | √ Yes   - Partial Yes - No - Includes only RCTs   **luded in the review?** | | |
|  | | For Yes  √ Must have reported on the sources of funding for individual studies included √ Yes in the review. Note: Reporting that the reviewers looked for this information  No but it was not reported by study authors also qualifies | | | | | | | |
| **11. If meta-analysis was performed did the review authors use appropriate methods for statistical combination of results?** | | | | | | | | | |
|  | **RCTs**  For Yes:  √ The authors justified combining the data in a meta-analysis  √ AND they used an appropriate weighted technique to combine study results and adjusted for heterogeneity if present.  √ AND investigated the causes of any heterogeneity | | | | √ Yes   - No - No meta-analysis conducted | | | |  |
|  | **For NRSI**  For Yes:  √ The authors justified combining the data in a meta-analysis  √ AND they used an appropriate weighted technique to combine study results, adjusting for heterogeneity if present  √ AND they statistically combined effect estimates from NRSI that were adjusted for confounding, rather than combining raw data, or justified combining raw data when adjusted effect estimates were not available   - - AND they reported separate summary estimates for RCTs and NRSI separately when both were included in the review | | | | √ Yes   - No - No meta-analysis conducted | | | |  |
| **12. If meta-analysis was performed, did the review authors assess the potential impact of RoB in individual studies on the results of the meta-analysis or other evidence synthesis?** | | | | | | | | | |
|  | For Yes:   - included only low risk of bias RCTs   √ OR, if the pooled estimate was based on RCTs and/or NRSI at variable RoB, the authors performed analyses to investigate possible impact of RoB on summary estimates of effect. | | | | √ Yes   - No - No meta-analysis conducted | | | |  |
| **13. Did the review authors account for RoB in individual studies when interpreting/ discussing the results of the review?** | | | | | | | | | |
|  | For Yes:   - included only low risk of bias RCTs   √ OR, if RCTs with moderate or high RoB, or NRSI were included the review provided a discussion of the likely impact of RoB on the results | | | | √ Yes   - No | | | |  |
| **14. Did the review authors provide a satisfactory explanation for, and discussion of, any heterogeneity observed in the results of the review?** | | | | | | | | | |
|  | For Yes:   - There was no significant heterogeneity in the results   √ OR if heterogeneity was present the authors performed an investigation of sources of any heterogeneity in the results and discussed the impact of this on the results of the review | | | | √ Yes   - No | | | |  |
| **15. If they performed quantitative synthesis did the review authors carry out an adequate investigation of publication bias (small study bias) and discuss its likely impact on the results of the review?** | | | | | | | | | |
|  | For Yes:  √ performed graphical or statistical tests for publication bias and discussed the likelihood and magnitude of impact of publication bias | | | | √ Yes   - No - No meta-analysis conducted | | | |  |
| **16. Did the review authors report any potential sources of conflict of interest, including any funding they received for conducting the review?** | | | | | | | | | |
|  | For Yes:  √ The authors reported no competing interests OR   - The authors described their funding sources and how they managed potential conflicts of interest | | | √ Yes   - No | | | | |  |

**Supplemental Table 3.** Definite search

| **Database** | **Definite search strategy** | **No. of articles** |
| --- | --- | --- |
| **Pubmed** | #1  (("Decision Support Systems, Management"[Mesh]) OR (((((((((Decision Support Systems, Clinical[MeSH Terms]) OR (Clinical Decision Support Systems[Title/Abstract])) OR (Clinical Decision Support System[Title/Abstract])) OR (Clinical Decision Support[Title/Abstract])) OR (Clinical Decision Supports[Title/Abstract])) OR (Decision Supports, Clinical[Title/Abstract])) OR (Support, Clinical Decision[Title/Abstract])) OR (Supports, Clinical Decision[Title/Abstract])) OR (Decision Support, Clinical[Title/Abstract])))  AND  #2  (((((((((((((((((((surgery[MeSH Terms]) OR (surgical procedures, operative[MeSH Terms])) OR (intraoperative complications[MeSH Terms])) OR (anesthesia[MeSH Terms])) OR (Perioperative Care[MeSH Terms])) OR (Perioperative Nursing[MeSH Terms])) OR (Perioperative Medicine[MeSH Terms])) OR (Surgery[Title/Abstract])) OR (operative[Title/Abstract])) OR (peroperative[Title/Abstract])) OR (perioperative[Title/Abstract])) OR (preoperative[Title/Abstract])) OR (postoperative[Title/Abstract])) OR (intraoperative[Title/Abstract])) OR (anesthesia[Title/Abstract])) OR (anaesthesia[Title/Abstract])) OR (Period, Perioperative[Title/Abstract])) OR (Periods, Perioperative[Title/Abstract])) OR (Perioperative Periods[Title/Abstract])) | 1309 |
| **MEDLINE** | #1  TS=("Decision Support Systems, Management" OR “Decision Support Systems, Clinical” OR “Clinical Decision Support Systems” OR “Clinical Decision Support System” OR “Clinical Decision Support” OR “Clinical Decision Supports” OR “Decision Supports, Clinical” OR “Support, Clinical Decision” OR “Supports, Clinical Decision” OR “Decision Support, Clinical”)  AND  #2  TS=(Surgery or operative or peroperative or perioperative or preoperative or postoperative or intraoperative or anesthesia or anaesthesia or “Period*, Perioperative” or “Perioperative Period*” or “general surgery” or “surgical procedures, operative” or “Perioperative Care” or “Perioperative Nursing” or “Perioperative Medicine”) | 1044 |
| **Embase** | #1  ("Decision Support Systems, Management" OR “Decision Support Systems, Clinical” OR “Clinical Decision Support Systems” OR “Clinical Decision Support System” OR “Clinical Decision Support” OR “Clinical Decision Supports” OR “Decision Supports, Clinical” OR “Support, Clinical Decision” OR “Supports, Clinical Decision” OR “Decision Support, Clinical”).ab,kf,kw,ti.  AND  #2  (Surgery or operative or peroperative or perioperative or preoperative or postoperative or intraoperative or anesthesia or anaesthesia or “Period*, Perioperative” or “Perioperative Period*” or “general surgery” or “surgical procedures, operative” or “Perioperative Care” or “Perioperative Nursing” or “Perioperative Medicine”).ab,kf,kw,ti. | 764 |
| **Web of Science** | #1  TS=("Decision Support Systems, Management" OR “Decision Support Systems, Clinical” OR “Clinical Decision Support Systems” OR “Clinical Decision Support System” OR “Clinical Decision Support” OR “Clinical Decision Supports” OR “Decision Supports, Clinical” OR “Support, Clinical Decision” OR “Supports, Clinical Decision” OR “Decision Support, Clinical”)  AND  #2  TS=(Surgery or operative or peroperative or perioperative or preoperative or postoperative or intraoperative or anesthesia or anaesthesia or “Period*, Perioperative” or “Perioperative Period*” or “general surgery” or “surgical procedures, operative” or “Perioperative Care” or “Perioperative Nursing” or “Perioperative Medicine”) | 663 |
| **Cochrane library** | #1  ("Decision Support Systems, Management" OR “Decision Support Systems, Clinical” OR “Clinical Decision Support Systems” OR “Clinical Decision Support System” OR “Clinical Decision Support” OR “Clinical Decision Supports” OR “Decision Supports, Clinical” OR “Support, Clinical Decision” OR “Supports, Clinical Decision” OR “Decision Support, Clinical”):ti,ab,kw  AND  #2  (Surgery or operative or peroperative or perioperative or preoperative or postoperative or intraoperative or anesthesia or anaesthesia or “Period, Perioperative” or “Perioperative Period” or “general surgery” or “surgical procedures, operative” or “Perioperative Care” or “Perioperative Nursing” or “Perioperative Medicine”):ti,ab,kw | 129 |

**Supplemental Text 1.** Reference List of Included Studies

1. Ali, U., et al., Reducing postoperative pain in children undergoing strabismus surgery: From bundle implementation to clinical decision support tools. Pediatric Anesthesia, 2020. 30(4): p. 415-423.

2. Colletti, A.A., et al., Feasibility and indicator outcomes using computerized clinical decision support in pediatric traumatic brain injury anesthesia care. Paediatr Anaesth, 2019. 29(3): p. 271-279.

3. Dherte, P.M., et al., Smart Alerts: Development of Software to Optimize Data Monitoring. Revista Brasileira De Anestesiologia, 2011. 61(1): p. 72-80.

4. Gabel, E., et al., Digital Quality Improvement Approach Reduces the Need for Rescue Antiemetics in High-Risk Patients: A Comparative Effectiveness Study Using Interrupted Time Series and Propensity Score Matching Analysis. Anesth Analg, 2019. 128(5): p. 867-876.

5. Gopwani, S., et al., Efficacy of Electronic Reminders in Increasing the Enhanced Recovery After Surgery Protocol Use During Major Breast Surgery: Prospective Cohort Study. JMIR Perioper Med, 2023. 6: p. e44139.

6. Gruss, C.L., et al., Automated feedback modestly improves perioperative treatment adherence of postoperative nausea and vomiting. J Clin Anesth, 2023. 86: p. 111081.

7. Gupta, R.K., Using an electronic clinical decision support system to reduce the risk of epidural hematoma. Am J Ther, 2014. 21(5): p. 327-30.

8. Hand, W.R., et al., Effect of a Cognitive Aid on Adherence to Perioperative Assessment and Management Guidelines for the Cardiac Evaluation of Noncardiac Surgical Patients. Anesthesiology, 2014. 120(6): p. 1339-1353.

9. Joosten, A., et al., Computer-assisted Individualized Hemodynamic Management Reduces Intraoperative Hypotension in Intermediate- and High-risk Surgery: A Randomized Controlled Trial. Anesthesiology., 2021. 05.

10. Kheterpal, S., A. Shanks, and K.K. Tremper, Impact of a Novel Multiparameter Decision Support System on Intraoperative Processes of Care and Postoperative Outcomes. Anesthesiology, 2018. 128(2): p. 272-282.

11. Kiatchai, T., et al., Development and Feasibility of a Real-Time Clinical Decision Support System for Traumatic Brain Injury Anesthesia Care. Appl Clin Inform, 2017. 8(1): p. 80-96.

12. Kooij, F.O., et al., Automated reminders increase adherence to guidelines for administration of prophylaxis for postoperative nausea and vomiting. Eur J Anaesthesiol, 2010. 27(2): p. 187-91.

13. Kooij, F.O., et al., Automated reminders decrease postoperative nausea and vomiting incidence in a general surgical population. British Journal of Anaesthesia, 2012. 108(6): p. 961-965.

14. Lakha, S., et al., Intraoperative Electronic Alerts Improve Compliance With National Quality Program Measure for Perioperative Temperature Management. Anesthesia and Analgesia, 2020. 130(5): p. 1167-1175.

15. Li, G., et al., The Impact of an Intraoperative Clinical Decision Support Tool to Optimize Perioperative Glycemic Management. Journal of Medical Systems, 2020. 44(10).

16. Lipps, J., et al., Physiologically Triggered Digital Cognitive Aid Facilitates Crisis Management in a Simulated Operating Room A Randomized Controlled Study. Simulation in Healthcare-Journal of the Society for Simulation in Healthcare, 2017. 12(6): p. 370-376.

17. McCormick, P.J., et al., Effectiveness of an Electronic Alert for Hypotension and Low Bispectral Index on 90-day Postoperative Mortality A Prospective, Randomized Trial. Anesthesiology, 2016. 125(6): p. 1113-1120.

18. McEvoy, M.D., et al., A Smartphone-based Decision Support Tool Improves Test Performance Concerning Application of the Guidelines for Managing Regional Anesthesia in the Patient Receiving Antithrombotic or Thrombolytic Therapy. Anesthesiology, 2016. 124(1): p. 186-98.

19. Mendez, J.A., et al., Improving the anesthetic process by a fuzzy rule based medical decision system. Artif Intell Med, 2018. 84: p. 159-170.

20. Nair, B.G., et al., Intraoperative blood glucose management: impact of a real-time decision support system on adherence to institutional protocol. Journal of Clinical Monitoring and Computing, 2016. 30(3): p. 301-312.

21. Nair, B.G., et al., Anesthesia Information Management System- Based Near Real-Time Decision Support to Manage Intraoperative Hypotension and Hypertension. Anesthesia and Analgesia, 2014. 118(1): p. 206-214.

22. Nair, B.G., et al., Near real-time notification of gaps in cuff blood pressure recordings for improved patient monitoring. J Clin Monit Comput, 2013. 27(3): p. 265-71.

23. Nair, B.G., et al., Feedback Mechanisms Including Real-Time Electronic Alerts to Achieve Near 100% Timely Prophylactic Antibiotic Administration in Surgical Cases. Anesthesia and Analgesia, 2010. 111(5): p. 1293-1300.

24. Nair, B.G., et al., Reducing wastage of inhalation anesthetics using real-time decision support to notify of excessive fresh gas flow. Anesthesiology, 2013. 118(4): p. 874-84.

25. Nellis, J.R., et al., A Risk-Prediction Platform for Acute Kidney Injury and 30-Day Readmission After Colorectal Surgery. J Surg Res, 2023. 292: p. 91-96.

26. Olmos, A.V., et al., Reducing Volatile Anesthetic Waste Using a Commercial Electronic Health Record Clinical Decision Support Tool to Lower Fresh Gas Flows. Anesth Analg, 2023. 136(2): p. 327-337.

27. Olsen, R.M., et al., Towards an automated multimodal clinical decision support system at the post anesthesia care unit. Comput Biol Med, 2018. 101: p. 15-21.

28. Parks, D.A., et al., Improving Adherence to Intraoperative Lung-Protective Ventilation Strategies Using Near Real-Time Feedback and Individualized Electronic Reporting. Anesth Analg, 2021. 132(5): p. 1438-1449.

29. Pouliot, J.D., et al., The Role of Computerized Clinical Decision Support in Reducing Inappropriate Medication Administration During Epidural Therapy. Hospital Pharmacy, 2018. 53(3): p. 170-176.

30. Pregnall, A.M., et al., Use of provider education, intra-operative decision support, and an email-feedback system in improving compliance with sugammadex dosage guideline and reducing drug expenditures. Journal of Clinical Anesthesia, 2022. 77 (no pagination).

31. Rinehart, J., et al., Intraoperative Stroke Volume Optimization Using Stroke Volume, Arterial Pressure, and Heart Rate: Closed-Loop (Learning Intravenous Resuscitator) Versus Anesthesiologists. Journal of Cardiothoracic and Vascular Anesthesia, 2012. 26(5): p. 933-939.

32. Shah, A.C., et al., Process Optimization and Digital Quality Improvement to Enhance Timely Initiation of Epidural Infusions and Postoperative Pain Control. Anesth Analg, 2019. 128(5): p. 953-961.

33. Shear, T.D., et al., The Effect of an Electronic Dynamic Cognitive Aid Versus a Static Cognitive Aid on the Management of a Simulated Crisis: A Randomized Controlled Trial. Journal of Medical Systems, 2019. 43(1).

34. St Pierre, M., et al., The effect of an electronic cognitive aid on the management of ST-elevation myocardial infarction during caesarean section: a prospective randomised simulation study. Bmc Anesthesiology, 2017. 17.

35. Velagapudi, M., et al., Evaluation of machine learning models as decision aids for anesthesiologists. Journal of Clinical Monitoring and Computing, 2023. 37(1): p. 155-163.

36. Wanderer, J.P., et al., Comparing two anesthesia information management system user interfaces: a usability evaluation. Can J Anaesth, 2012. 59(11): p. 1023-31.

37. Wetmore, D., et al., An embedded checklist in the Anesthesia Information Management System improves pre-anaesthetic induction setup: a randomised controlled trial in a simulation setting. Bmj Quality & Safety, 2016. 25(10): p. 739-746.

38. Wissel, B.D., et al., Automated, machine learning-based alerts increase epilepsy surgery referrals: A randomized controlled trial. Epilepsia, 2023. 64(7): p. 1791-1799.

39. Zaouter, C., et al., A novel system for automated propofol sedation: hybrid sedation system (HSS). J Clin Monit Comput, 2017. 31(2): p. 309-317.

40. Zaouter, C., et al., Use of a decision support system improves the management of hemodynamic and respiratory events in orthopedic patients under propofol sedation and spinal analgesia: a randomized trial. J Clin Monit Comput, 2014. 28(1): p. 41-7.
